# Development and Implementation of a Double-Blind Corticosteroid-Tapering Regimen for a Clinical Trial

**DOI:** 10.1155/2015/589841

**Published:** 2015-03-24

**Authors:** Neil Collinson, Katie Tuckwell, Frank Habeck, Monique Chapman, Micki Klearman, John H. Stone

**Affiliations:** ^1^Roche Products Ltd., Welwyn Garden City AL7 1TW, UK; ^2^F. Hoffmann-La Roche, 4070 Basel, Switzerland; ^3^Genentech, South San Francisco, CA 94080, USA; ^4^Massachusetts General Hospital, Harvard Medical School, Boston, MA 02114, USA

## Abstract

We describe the design and operationalization of a blinded corticosteroid-tapering regimen for a randomized trial of tocilizumab in giant cell arteritis (GCA). To our knowledge, no clinical trial in any disease has ever employed a blinded corticosteroid-tapering regimen, but this was necessary to the design of our trial which is likely to be relevant to other investigations of steroid-sparing regimens. Two standardized corticosteroid-tapering regimens are required for this GCA trial: a 6-month regimen in 3 arms (taken with tocilizumab 162 mg subcutaneously weekly or every other week or with placebo) and a 12-month regimen with placebo (fourth arm). Investigators select initial prednisone doses, tapered in an open-label fashion until 20 mg/day. Doses <20 mg/day are blinded. At least 27 blinded blister packs are required to ensure blinding and encourage compliance. This permits all possible daily doses but requires ≤5 capsules/day. The number of capsules taken at any point during tapering is identical across groups. Our approach may be extrapolated to trials beyond GCA.

## 1. Introduction

Corticosteroids, used either alone or in combination with other immunosuppressive medications, remain a cornerstone of treatment for many inflammatory diseases. Corticosteroid use crosses multiple subspecialty borders. These medications, highly effective for many conditions, are regarded generally as the anti-inflammatory agents with the swiftest onset of effect. For some diseases, such as giant cell arteritis (GCA), corticosteroids are the only type of medication known to be effective.

A broad paradigm for the treatment of inflammatory disease involves a strategy that initially employs high doses of corticosteroid sufficient to control acute inflammation, followed by tapering—usually over months—to lower daily doses when possible. A significant percentage of patients in many diseases, however, are unable to discontinue corticosteroid use because of disease recurrence [1, 2]. Although corticosteroids are efficacious, they are regarded by physicians and patients alike as a “double-edged sword” because of their association with a broad spectrum of adverse effects [1, 3, 4]. Such adverse effects include hypertension, glucose intolerance, osteoporosis, cataracts, gastrointestinal bleeding, proximal muscle weakness, skin thinning, psychosis, depression, heightened risk for infection, and a decrease in the overall quality of life [1, 3–5].

The development of a range of noncorticosteroid therapies that are effective in some immune-mediated diseases offers the possibility of reducing dependency on corticosteroids. Agents that have a steroid-sparing effect would be a welcome addition to the current treatment armamentarium. However, the rigorous demonstration of steroid-sparing effects involves the conduct of randomized, double-blind trials. Herein lies a significant hurdle: to our knowledge, no clinical trial involving the double-blind administration of a variable-dose, variable-tapering rate corticosteroid regimen has ever been performed. Consequently, the logistical issues for designing, operationalizing, and conducting such a trial have never been undertaken.

GCA, a form of vasculitis that affects large and medium-sized vessels, is characterized by headaches, ischemia-related visual manifestations (including blindness), polymyalgia rheumatica, claudication of the jaw and extremities, constitutional symptoms, myocardial infarction, and stroke [6]. Corticosteroids are highly effective at controlling systemic inflammation and preventing acute ischemic damage but are less successful at maintaining remission. Between 50% and 80% of patients experience relapses during dose reduction and require long-term corticosteroid courses [1, 6].

GiACTA is a multicenter, randomized, double-blind, placebo-controlled trial designed to evaluate the ability of tocilizumab to maintain disease remission and to reduce cumulative corticosteroid exposure in patients with GCA (ClinicalTrials.gov, number NCT01791153) [7]. The trial design required not only the blinding of tocilizumab but also the blinding of corticosteroid regimens of 2 different durations used in the trial. We describe here the challenges inherent in the design of a clinical trial involving blinded corticosteroid regimens and outline our approach to the development and operationalization of the blinded corticosteroid-tapering regimen used in the GiACTA trial.

## 2. Methods

### 2.1. Overview of the GiACTA Trial

**Figure 1:**
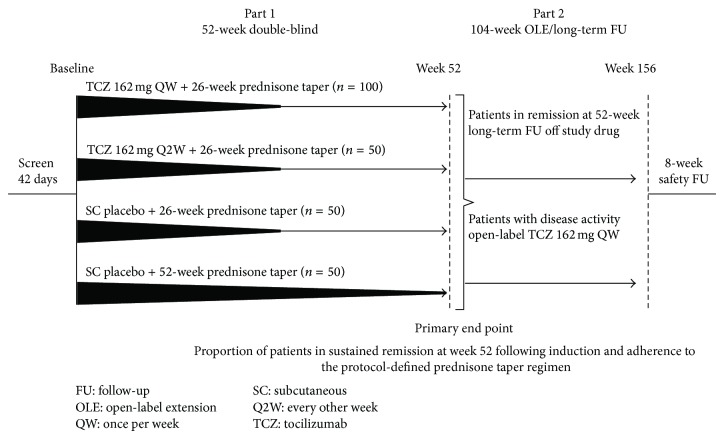
GiACTA study design featuring standardized prednisone-tapering protocols.

The GiACTA trial design has been published [7]. The trial population, when complete, will consist of approximately 250 patients (as of December 1, 2014, 192 patients were enrolled). Enrolled patients are assigned randomly to 1 of 4 groups (Figure 1). Two of these groups receive tocilizumab at different doses plus prednisone (6-month tapers), and the other 2 receive prednisone alone with tapering regimens of either 6 or 12 months. The treatment regimens in the 4 arms are as follows: arm A, subcutaneous tocilizumab 162 mg/week, combined with a 6-month corticosteroid taper; arm B, subcutaneous tocilizumab 162 mg every other week, combined with a 6-month corticosteroid taper; arm C, prednisone alone, 6-month taper; arm D, prednisone alone, 12-month taper [7]. Patients in arms C and D receive subcutaneous tocilizumab placebo injections.

### 2.2. Institutional Review Board Approval and Informed Consent

Each participating clinic will have institutional review board oversight. All participants will give written informed consent.

### 2.3. Prednisone Regimen in GiACTA

Corticosteroids are administered in GiACTA according to a protocol-defined prednisone-tapering schedule. The 6-month taper is designed to test the ability of tocilizumab to maintain disease remission at 1 year after the discontinuation of corticosteroids. Because the optimal duration of corticosteroids in GCA is unknown, an arm with a 12-month CS taper is also included. The 6- and 12-month corticosteroid-tapering regimens approximate closely those used in many inflammatory diseases, balancing the goals of controlling active inflammation quickly but tapering and eventually discontinuing prednisone within a reasonable time frame to prevent potentially unnecessary corticosteroid-induced adverse effects.

### 2.4. Identifying the Challenges Confronting Development of a Blinded Prednisone Taper

Translating complex blinded corticosteroid-tapering regimens to a format that could be understood easily and followed reliably by both study site staff and patients posed a number of challenges. First, the final product had to be simple enough to be operationalized by more than 100 clinical trial sites across North America and Europe, covering 15 different languages. The prednisone/prednisone-placebo tablets had to be sufficiently user-friendly so that patients could take the medication in compliance with the protocol.

Second, patients are entering the trial at different prednisone doses, based on the initial dose which the investigator believes is necessary to control the disease. The dose could range from 20 mg/day to a maximum of 60 mg/day. Furthermore, maintenance of the trial blind required that the number of pills taken must be the same across all 4 arms on any day of the taper, regardless of whether patients are assigned to the 6-month or the 12-month taper.

Third, prednisone tablets are manufactured in multiple denominations, including 1 mg, 1.5 mg, 2.5 mg, 5 mg, 10 mg, 20 mg, and 60 mg tablets. Creating identical placebos for all doses would have substantially increased the cost of the blinding process. In addition, the plan ultimately used would have had to minimize the number of tablets any patient was required to take on the same day. Finally, a system for measuring compliance with the prednisone regimen had to be created, and mitigation plans for tablet loss and noncompliance had to be developed to ensure minimal impact on a patient's tapering regimen.

## 3. Results

### 3.1. Fundamental Decision about Prednisone Taper

**Figure 2:**
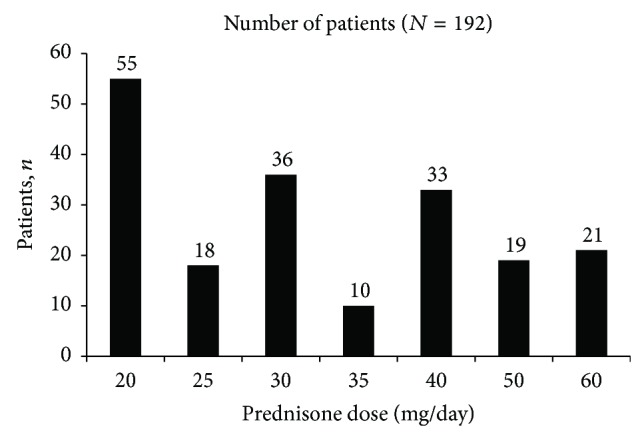
Initial prednisone dose of patients entering the trial.

Each patient enters the trial on an open-label prednisone dose between 20 and 60 mg/day, chosen by the investigator based on disease presentation. The initial prednisone dose selected for the first 192 patients, shown in Figure 2, illustrates the range of doses at entry.

The blinded portion of the prednisone taper does not begin until the patient tapers to below 20 mg/day. This permits GCA patients who have active disease to be treated immediately with corticosteroids, even as preparations are made to enroll them in the trial. To permit an unbiased evaluation of the corticosteroid-sparing effects of tocilizumab, however, patients and investigators are blinded to the prednisone taper schedule below 20 mg/day.

### 3.2. Use of Blister Packs

**Figure 3:**
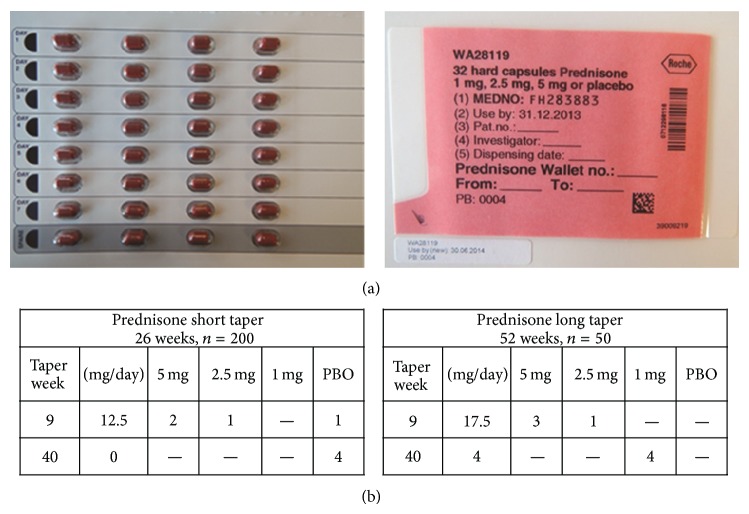
(a) Final prednisone wallets developed and approved for the GiACTA study. (b) Illustration of the use of placebo capsules to ensure blinding. Two separate weeks from the six- and 12-week prednisone tapering regimens are shown. Placebo capsules are used to ensure that patients receive an equal number of capsules across both regimens, thus ensuring blinding. PBO: placebo.

Blinding is achieved by the provision of encapsulated prednisone/placebo doses in numbered blister packs (Figure 3). The total duration and cumulative dose of prednisone therapy for each patient depend on the initial dose at entry into the trial and on the treatment arm to which the patient is assigned. The daily dose comprises active prednisone capsules, prednisone-placebo capsules, or a combination of both.

### 3.3. Development of Prednisone Packaging

Overencapsulation of commercially available prednisone tablets is used to preserve the trial blind. The prednisone manufacturer (Roxane Laboratories Inc., Columbus, OH) was selected based on acceptance by regulatory authorities and commercial availability. Regulations require that tablets be an acceptable size for swallowing and meet certain dissolution criteria after overencapsulation. Each capsule contains 1 prednisone tablet along with filling material to secure the tablet within the capsule. Placebo capsules contain only filling material but weigh approximately the same as those containing prednisone. The protocol-mandated tapering regimens are achieved using combinations of only 3 denominations of encapsulated prednisone tablets—1 mg, 2.5 mg, and 5 mg—and the placebo capsules.

Generic prednisone tablets were overencapsulated at a contract manufacturing organization (Fisher Clinical Services Inc., Center Valley, PA). To gain stability data on the capsules, technical prednisone batches without intention for use in humans were manufactured and placed in the selected primary packaging material (duplex-blister packs). After the first analysis of each technical batch (1 mg, 2.5 mg, and 5 mg prednisone and placebo formulations), Roche Regulatory Affairs was able to generate an Investigational Medicinal Product Dossier for the Clinical Trial Application. The same approach was applied to batches intended for use in humans. Strict adherence to Good Manufacturing Practice was followed throughout the process.

### 3.4. Uniformity across the 2 Tapering Regimens

The tapering scheme is designed in weekly decrements. Capsules required for any given week are placed between 1 and 5 vertical blister strips on a single card held within a wallet containing all the capsules required for 1 week. The 7 horizontal rows are organized according to day, and each card has a spare row to be used in the event a capsule is lost. To ensure blinding, the number of capsules taken at any given point is identical across the 6- and 12-month prednisone-tapering regimens. Multiple weekly blister pack combinations (27 in all) are designed to maximize convenience for patients and to foster compliance. This approach ensures that the prednisone dose is typically 3 but never more than 5 capsules in a single day.

### 3.5. Distribution of Prednisone Packs to Sites

An interactive voice response system (IVRS) is used to distribute the appropriate prednisone packs from depots to sites, ready for the patient to collect at each study visit. At each monthly study visit, patients are given 4 blinded steroid wallets, each of which is to last 1 week. Each blister pack bears a unique alphanumeric identifier that allows the IVRS to instruct the sites on allocation of the appropriate blister packs. The identifier also indicates the order in which the steroid wallets are to be used. During dispensing, study staff can write additional patient instructions on the prednisone wallets for the sake of clarity. Figure 3 shows the final prednisone wallets developed and approved by the Quality Department at Roche.

### 3.6. Training of Trial Site Staff

At investigators' meetings held in Boston and Berlin before enrollment began, the study staff at each clinical site was trained on the corticosteroid-tapering regimens and the approach to blinding. The training consisted of video and lecture components and allowed the staff to gain hands-on familiarity with the blister packs, the wallets, and the overall plan for dispensing corticosteroids.

### 3.7. Patient and Investigator Guidance Documents

To facilitate investigator and patient comprehension of blister pack use and to foster compliance, illustrated guidance documents and patient diaries were provided (Table 1). Study site staff received a booklet that provided guidance on prednisone dispensation and administration and that described the correct way to track dispensation and administration on the electronic case report form. At enrollment, patients receive a booklet that aids in their understanding of the prednisone packaging design, offers instructions on compliance, and provides problem-solving scenarios regarding blister pack use.

### 3.8. Experience with Blinded Corticosteroid Tapering in the Trial to Date

As the GiACTA study has progressed, sites and patients have become familiar with the technical and logistical issues of the blinded corticosteroid regimens. Among the first 170 patients enrolled, errors have typically occurred during the first few weeks after enrollment and have consisted of patients selecting the incorrect blister pack (*n* = 2), taking an incorrect number of capsules/tablets (*n* = 1), missing medication because of vacation (*n* = 1), initiating a new dose on an incorrect day (*n* = 10), and incorrectly recording medication intake (*n* = 13). None of the errors has resulted in a major protocol deviation or necessitated discontinuation of the corticosteroid regimen.

## 4. Discussion

The field of rheumatology and the treatment of all inflammatory diseases were revolutionized more than 6 decades ago by the discovery of cortisone as a therapeutic agent for rheumatoid arthritis [8]. Since then, corticosteroids have been used extensively in essentially every specialty of medicine and surgery for the treatment of patients with inflammatory conditions. Corticosteroids have proven to be life-saving drugs in many diseases. In patients with GCA, for example, the administration of corticosteroids as soon as the diagnosis is suspected contributes powerfully to initial control of the disease and preservation of vision, the loss of which is patients' greatest fear. Nevertheless, the adverse effects associated with prolonged corticosteroid use, even in relatively low doses, are substantial, and the side effects associated with the long-term use of moderate doses are unacceptable. Indeed, corticosteroid-associated morbidity is viewed by patients as the single greatest impediment to good quality of life [5].

With the advent of the biologic era of therapeutics for inflammatory diseases, it is now conceivable that the need for long-term reliance on corticosteroids to maintain disease control can be obviated in many conditions. In kidney transplantation, for example, a common goal now is to achieve posttransplantation courses that are corticosteroid-free after several weeks, particularly in the case of living-related donor transplantation [9]. The development of “steroid-free” remissions and the identification of new medications that enable steroid-sparing approaches to treatment are important goals in managing immune-mediated conditions. The most rigorous manner of assessing the steroid-sparing ability of a medication is through the conduct of randomized clinical trials that make use of blinded corticosteroid regimens.

Several lessons from our approach can be extrapolated to trials in other immune-mediated conditions in which corticosteroid sparing is a therapeutic requirement, thereby helping to address an important need in the area of inflammation. First, blinded corticosteroid tapers are feasible in clinical trials, even in the setting of complex treatment protocols that involve the blinding of an experimental medication (in this case, tocilizumab). Second, depending on the specific features of the disease under study and the clinical trial protocol, blinding of all corticosteroid doses may not be necessary. In GiACTA, the decision to blind prednisone doses only below 20 mg/day was crucial to the implementation of an effective design. Trials of other medications in other inflammatory diseases may emulate this approach because the ability of corticosteroids to control most inflammatory diseases does not wane in most conditions until patients reach doses lower than 20 mg/day. Third, systematic programs for the education of trial staff and patients about the use of the prednisone-blinding methodology chosen are essential to the success of such trials.

To our knowledge, this is the first clinical trial in any disease to use blinded, variable-dose, variable-duration corticosteroid regimens. Our approach permitted us to overcome significant technical and operational challenges to fulfill the requirements of a complex clinical trial protocol. The creative approaches to solving the logistical problems are necessary to ensure an unbiased evaluation of the ability of tocilizumab to serve as a steroid-sparing treatment in GCA. Adaptations of this approach may find broad use in other trials designed to investigate the steroid-sparing effect of new medications and to limit the morbidity from corticosteroids endured by patients with inflammatory conditions.

## Figures and Tables

**(a):** 

	Prednisone dispensed at visit					When medication dispensed should be used by patient						
Visit name	(Additional prednisone may be dispensed at some study visits to allow for the maximum study visit window and duration between study visits)		IxRS reference	eCRF reference		Patient diary reference		Prednisone	Prednisone taken by patient at corresponding patient diary study week number			
	Type	Comments		Visit folder	Study week number	Study week number	Wallet number^∗^ (taper week)^∗^ blinded part only		Type	Number per day	Number of tablets remaining	Medication returned at end of 7 days
Baseline	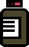	1 open label 10 mg bottle containing 100 tablets	Baseline to study week 1	Baseline	0	0	1	Open-label taper 60 mg/d	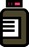	6 tablets per day	58 tablets remaining	No continue to use tablets in study week 1
			Study week 1 to study week 2	Week 1	1	1	2	Open-label taper 50 mg/d		5 tablets per day	23 tablets remaining	Yes return at study week 2 visit

Study week 1	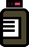	1 open label 10 mg bottle containing 100 tablets	Study week 2 to study week 3	Week 2	2	2	3	Open-label taper 40 mg/d	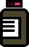	4 tablets per day	72 tablets remaining	Yes return at study week 3 visit

Study week 2	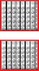	2 open label 5 mg wallet (wallet contains 40 capsules)	Study week 3 to study week 4	Week 3	3	3	4	Open-label taper 35 mg/d	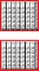	7 capsules per day	31 capsules remaining	No continue to use capsules in study week 4

**(b):** 

Record of steroid medication capsules	Blinded steroids		Part 1 of the study

Study week number	Wallet number/date wallet started dd/mm/yyyy	Were any capsules missed?	Comments

—	Prednisone Wallet Number_________	No_____ Yes_____	*Please provide the reasons for any capsules missed and any other information about your steroid medication this week*.
		*If Yes, please provide the number of capsules that were missed this week*.	
	Date started ___/___/___	___________capsule(s) missed	_____________________________________ _____________________________________

—	Prednisone Wallet Number_______________	No____ Yes____	*Please provide the reasons for any capsules missed and any other information about your steroid medication this week*.
		*If Yes, please provide the number of capsules that were missed this week*.	
	Date started ___/___/___	___________capsule(s) missed	_____________________________________ _____________________________________

Record of steroid medication tablets	Open label 60 mg steroids/per day		Part 1 of the Study

Study week number	Date weekly tablets started dd/mm/yyyy	Were any tablets missed?	Comments

—	Prednisone Wallet Number_________	No___ Yes___	*Please provide the reasons for any tablets missed and any other information about your steroid medication this week*.
		*If Yes, please provide the number of tablets that were missed this week*.	
	Date started ___/___/___	________tablet(s) missed	______________________________________ ______________________________________
